# Differential DNA Methylation in Umbilical Cord Blood of Infants Exposed to Low Levels of Arsenic *in Utero*

**DOI:** 10.1289/ehp.1205925

**Published:** 2013-06-11

**Authors:** Devin C. Koestler, Michele Avissar-Whiting, E. Andres Houseman, Margaret R. Karagas, Carmen J. Marsit

**Affiliations:** 1 Department of Community and Family Medicine, Geisel School of Medicine, Dartmouth College, Hanover, New Hampshire, USA; 2 Department of Pathology and Laboratory Medicine, Brown University, Providence, Rhode Island, USA; 3 Department of Public Health, Oregon State University, Corvallis, Oregon, USA; 4 Department of Pharmacology and Toxicology, Geisel School of Medicine, Dartmouth College, Hanover, New Hampshire, USA

**Keywords:** arsenic, cord blood, DNA methylation, epigenetics, Illumina 450K, *in utero* arsenic exposure

## Abstract

Background: There is increasing epidemiologic evidence that arsenic exposure *in utero*, even at low levels found throughout much of the world, is associated with adverse reproductive outcomes and may contribute to long-term health effects. Animal models, *in vitro* studies, and human cancer data suggest that arsenic may induce epigenetic alterations, specifically by altering patterns of DNA methylation.

Objectives: In this study we aimed to identify differences in DNA methylation in cord blood samples of infants with *in utero*, low-level arsenic exposure.

Methods: DNA methylation of cord-blood derived DNA from 134 infants involved in a prospective birth cohort in New Hampshire was profiled using the Illumina Infinium Methylation450K array. *In utero* arsenic exposure was estimated using maternal urine samples collected at 24–28 weeks gestation. We used a novel cell mixture deconvolution methodology for examining the association between inferred white blood cell mixtures in infant cord blood and *in utero* arsenic exposure; we also examined the association between methylation at individual CpG loci and arsenic exposure levels.

Results: We found an association between urinary inorganic arsenic concentration and the estimated proportion of CD8^+^ T lymphocytes (1.18; 95% CI: 0.12, 2.23). Among the top 100 CpG loci with the lowest *p*-values based on their association with urinary arsenic levels, there was a statistically significant enrichment of these loci in CpG islands (*p* = 0.009). Of those in CpG islands (*n* = 44), most (75%) exhibited higher methylation levels in the highest exposed group compared with the lowest exposed group. Also, several CpG loci exhibited a linear dose-dependent relationship between methylation and arsenic exposure.

Conclusions: Our findings suggest that *in utero* exposure to low levels of arsenic may affect the epigenome. Long-term follow-up is planned to determine whether the observed changes are associated with health outcomes.

## Introduction

Arsenic, an established environmental toxicant, has been linked to numerous adverse health outcomes, including cardiovascular disease (Chen et al. 2011; Gong and O’Bryant 2012), cancer (Hopenhayn-Rich et al. 1998), and skin lesions (Yunus et al. 2011). There is also a growing concern regarding the effects of *in utero* exposure to arsenic on the developing fetus (Vahter 2008). Recent studies have reported that high-level exposure to arsenic *in utero* is associated with increased infant mortality, low birth weight, and birth defects (Rahman et al. 2009, 2010). Most previous studies of arsenic exposure and newborn health outcomes, however, have focused primarily on populations in arsenic-endemic regions, outside the United States (Vahter 2008). Whereas occupational exposure to arsenic and contaminated drinking water were once considered the primary means of arsenic exposure, dietary intake of arsenic, particularly from rice consumption, potentially may contribute to the same extent as drinking water in exposing pregnant women to arsenic (Gilbert-Diamond et al. 2011). This finding has raised concerns regarding the possible health consequences associated with fetal exposure to what may be relatively common levels of arsenic. Yet there exists a gap in current knowledge as to whether findings from populations in arsenic endemic regions of the world can be used to infer the risks associated with low-level arsenic exposure, common to much of the United States.

Emerging data now suggest that arsenic may induce epigenetic alterations, specifically by altering patterns of DNA methylation (Reichard and Puga 2010; Ren et al. 2011). Differences in global DNA methylation associated with exposure to arsenic have been reported based on animal (Davis et al. 2000; Zhao et al. 1997), *in vitro* (Mass and Wang 1997), and human studies across various tissue types (i.e., umbilical cord blood and peripheral blood in adult subjects) (Intarasunanont et al. 2012; Wilhelm et al. 2010). The patterns of DNA methylation alteration associated with arsenic exposure have been far from consistent, with several studies reporting reductions in global DNA methylation (Intarasunanont et al. 2012; Wilhelm et al. 2010; Zhao et al. 1997) and others reporting evidence of global hypermethylation (Davis et al. 2000; Mass and Wang 1997). Factors including the type of markers used for assessment of global methylation, the biological sample in which DNA methylation was measured, and modifiers such as nutritional folate levels (Pilsner et al. 2007) may explain the differential findings. In the context of prenatal exposure to arsenic, two recently published studies of different Bangladesh cohorts have reported positive associations between LINE-1 repeat element methylation in cord blood and arsenic exposure *in utero* (Kile et al. 2012; Pilsner et al. 2012), although no significant associations were observed in a cross-sectional study conducted in Thailand (Intarasunanont et al. 2012).

Beyond global methylation alterations, several recent epidemiologic studies have reported positive gene-specific associations between *in utero* exposure to arsenic and promoter methylation of *CDKN2A* (encoding p16INK4A) (Kile et al. 2012) and *TP53* (Intarasunanont et al. 2012) measured in human umbilical cord peripheral blood lymphocytes (PBLs). However, the reported associations of these two studies correspond to very modest differences in DNA methylation, even though these studies consisted of individuals residing in the areas of the world with the highest purported levels of arsenic exposure. These studies, as well as studies of adults, have measured DNA methylation in unfractioned PBLs, which are relatively easy to collect and process. However, the primary limitation is that methylation signatures in PBLs represent the aggregate methylation profile of a complex cellular mixture. Thus, even small changes in percent methylation may indicate considerable differences in underlying cell populations, reflecting immunomodulation. Arsenic exposure has been associated with immune suppression and impaired macrophage function in exposed populations (Banerjee et al. 2009; Selgrade 2007). Hence, one possible mechanism for arsenic-induced diseases in exposed populations is altered immune function related to alterations of immune cell populations, which may be evident in blood-based profiles of DNA methylation. Dissecting out the contributions of different cell types and direct changes to the methylome on the observed associations between arsenic exposure and gene-specific DNA methylation patterns is critical for understanding the mechanisms of arsenic’s immunotoxic effects. To address this limitation, our group has begun to use DNA methylation signatures of known cell types as a surrogate for defining cell mixture proportions (Houseman et al. 2012); by applying this methodology, we are able to interrogate not only arsenic’s effect on DNA methylation but also its effect on relative leukocyte subtype proportions.

Despite the collective evidence supporting the role of arsenic exposure on the dysregulation of DNA methylation, little is known about potential effects of low levels of arsenic *in utero*—common to much of the world’s population—on DNA methylation. Moreover, the extent to which such patterns reflect immunomodulation, indicated by shifts in leukocyte subpopulations, or represent changes in the underlying methylome, has not been previously examined. We aimed to address these questions by examining the association between low-level arsenic exposure at 24–28 weeks gestation, a period of exposure during which DNA methylation patterns are becoming set in hematopoetic stem cells (Rodak et al. 2007), and patterns of epigenome-wide DNA methylation in umbilical cord blood samples from 134 mother–infant pairs enrolled in a U.S.-based cohort study.

## Methods

*Study population*. The study population consisted of the 134 initial participants of the ongoing New Hampshire Birth Cohort Study (NHBCS), which focuses on pregnant women from New Hampshire, whose primary household drinking-water source was a private well (Gilbert-Diamond et al. 2011). Eligibility criteria included English speaking, English literate, and mentally competent pregnant women 18–45 years of age. Subjects who changed their residence since their last menstrual period or whose home water supply was from a source other than from a private well were excluded from the study. Demographic and lifestyle information was collected during routine prenatal visits, and for the infant from the newborn medical chart. This study was approved by the Committee for the Protection of Human Subjects at Dartmouth College. All study participants provided written informed consent prior to the study.

*Arsenic exposure assessment*. As previously described (Gilbert-Diamond et al. 2011), spot urine samples were collected at approximately 24–28 weeks gestation into acid-washed containers that contained 30 μL of 10 mM diammonium diethyldithiocarbamate to stabilize arsenic species, and frozen at –80°C until analysis (within 24 hr of collection). Samples were analyzed for individual species of urinary arsenic using a high-performance liquid chromatography inductively coupled plasma mass spectrometry (ICP-MS) system, and urinary creatinine levels were assessed to control for urinary dilution. The arsenic speciation method is capable of quantitatively determining five arsenic species in urine: arsenite (As^III^), arsenate (As^V^), dimethylarsinic acid (DMA^V^), monomethylarsonic acid (MMA^V^), and arsenobetaine. The separated arsenic species were detected by ICP-MS using time-resolved analysis at *m*/*z* 75. The detection limits ranged from 0.10 to 0.15 μg/L for the individual arsenic species. Values for samples with measurements below the limit of detection were taken to be the median between 0 μg/L and the detection limit for that arsenic species. We calculated total urinary arsenic concentrations (U-As) by summing inorganic arsenic (iAs; As^III^ and As^V^) and the metabolic products MMA^V^ and DMA^V^. Arsenobetaine was excluded from this calculation because it is thought to be nontoxic and to pass through the body without being metabolized. We used total U-As as a measure of *in utero* exposure to arsenic because urinary arsenic levels have been suggested to provide reliable indications of internal dose (Marchiset-Ferlay et al. 2012), and arsenic is known to readily cross the placenta, leading to fetal serum concentrations similar to maternal levels (Concha et al. 1998). As a measure of methylation efficiency, we have also calculated the ratio of inorganic to total urinary arsenic [iAs/(iAs + MMA^V^ + DMA^V^)].

*DNA methylation assessment and quality control.* DNA was isolated from cord blood samples using DNeasy® blood & tissue kits (Qiagen, Valencia, CA) and bisulfite converted using the EZ DNA Methylation kit (Zymo, Irvine, CA). Samples were randomized across several plates and subsequently subjected to epigenome-wide DNA methylation assessment using the Illumina Infinium HumanMethylation450 BeadChip (Illumina, San Diego, CA), which simultaneously profiles the methylation status for > 485,000 CpG sites at single-nucleotide resolution. Microarrays were processed at the Biomedical Genomics Center at the University of Minnesota (Minneapolis, MN), following standard protocols. The methylation status for each individual CpG locus was calculated as the ratio of fluorescent signals (β = Max(M,0)/[Max(M,0) + Max(U,0) + 100]), ranging from 0 (no methylation) to 1 (complete methylation), using the average probe intensity for the methylated (M) and unmethylated (U) alleles. The data were assembled using BeadStudio methylation software (Illumina, San Diego, CA), without normalization per the manufacturer’s instructions. We used array control probes to assess the quality of our samples and evaluate potential problems such as poor bisulfite conversion or color-specific issues for each array (Marsit et al. 2009). All CpG loci on X and Y chromosomes and all loci within 100bp of known single-nucleotide polymorphisms (SNPs) (determined using the annotation for the Illumina HumanMethylation450 array) were excluded from the analysis to avoid sex-specific methylation bias and biases related to genetic variability, respectively, leaving 385,249 autosomal CpG loci for analysis in 134 samples. Technical validation of the methylation array measurements was obtained using bisulfite pyrosequencing [for details, see Supplemental Material, Bisulfite Pyrosequencing (http://dx.doi.org/10.1289/ehp.1205925)].

## Statistical Analysis

*Principal components analysis and adjustment for plate effects*. DNA methylation values were logit [i.e., log(β/1–β)] transformed as in previous studies (Du et al. 2010; Kuan et al. 2010). To de-convolve the most prevalent sources of variability in DNA methylation across the array, we performed a principal components analysis (PCA) on the resulting methylation data (Harper et al. 2013; Yang et al. 2010). PCA represents a feature extraction technique where the methylation data is orthogonally transformed, such that the first principal component has the largest possible variance (accounts for maximal amount of variability in the methylation data), and each succeeding component in turn has the next highest variance possible. The resulting top three principal components (those representing the maximum proportion of variability in methylation) were then examined in terms of their association with technical aspects concerning the array (i.e., plate/BeadChip) and patient demographic information using a series of linear regression models [see Supplemental Material, Figures S1–S2 and Table S1 (http://dx.doi.org/10.1289/ehp.1205925)]. Because the top three principal components were significantly associated with plate (see Supplemental Material, Figure S2, Table S1), suggesting that plate was a major source of variability in methylation across the array, we adjusted for plate effects by applying the ComBat method (Johnson et al. 2007). Following a similar procedure, we then investigated the resulting plate-adjusted methylation data to ensure that variation in methylation across the array induced by plate effects had been successfully attenuated (see Supplemental Material, Figure S2, Table S2). We also investigated the top three principal components computed from the plate-adjusted methylation data in terms of their association with arsenic exposure. Briefly, this was accomplished by fitting a series of linear regression models that modeled the principal component as the dependent variable and quartiles of U-As as an independent variable, which were adjusted for maternal age at delivery, infant sex, and urinary creatinine levels (Barr et al. 2005; Gamble and Liu 2005). Unless stated otherwise, quartiles of U-As were used as measures of arsenic exposure because several studies have reported nonlinear relationships between arsenic exposure and patterns of DNA methylation at both gene-specific and genome-wide levels (Chanda et al. 2006; Majumdar et al. 2010) and also to reduce any skewing of the data by outlying values.

*Cell mixture deconvolution analysis*. Using the plate-adjusted methylation data, we employed a novel statistical methodology (Houseman et al. 2012) for inferring changes in the distribution of leukocytes between quartiles of U-As using DNA methylation signatures, combined with a previously obtained external reference data set consisting of methylation signatures from purified leukocyte samples (Houseman et al. 2012; Koestler et al. 2012). Further details regarding the reference data set are provided elsewhere (Houseman et al. 2012; Koestler et al. 2012). A critical component of this approach is the set of DNA methylation signatures of the major leukocyte components of whole blood [i.e., B cells, natural killer (NK) cells, CD8^+^ T lymphocytes, CD4^+^ T lymphocytes, monocytes, and granulocytes]. The methods of Houseman et al. (2012) demonstrate that the distribution of white blood cells can be approximated from the DNA methylation measured in whole blood at the top 500 leukocyte differentially methylated regions (L-DMRs); application of these methods allowed us to estimate the expected difference in cell type proportions between U-As quartiles 2, 3, and 4 and the referent quartile (quartile 1), as well as the expected change in cell type proportions based on a 1-μg/L increase in the concentration of the individual arsenic metabolites.

In addition, the methods of Houseman et al. (2012) allowed us to quantify the proportion of total variability in cord blood DNA methylation explained by estimated immune cell composition. Consistent with our other models, we adjusted for maternal age at delivery, infant sex, and urinary creatinine levels. We note several assumptions regarding the cell mixture deconvolution method. First, we assumed that the 500 L-DMRs—discerned using the methylation signature from purified leukocyte subtypes from healthy adults—are indeed biologically determinant of key differences in cell type across all ages, and therefore translate to cord-blood. We also assumed that the methylation status for the 500 L-DMRs used here is not systematically altered by exposure to arsenic. We note other work (Koestler et al. 2013) that demonstrates the insensitivity of the methods to the deletion of some CpGs (e.g., those that show evidence of serving as age-DMRs)—that the accidental inclusion of some age-DMRs in the set are unlikely to influence results. Also, substantial biases due to arsenic exposure would require systematic alterations of methylation states at the 500 L-DMRs in a manner consistent with the linear space spanned by the reference profiles, an event unlikely unless the alteration was itself implicated in perturbations of hematopoiesis.

*Locus–locus analysis for detecting differentially methylated CpG loci*. We implemented a locus-by-locus analysis aimed toward identifying differentially methylated CpG sites based on total urinary arsenic levels. Briefly, analysis of covariance models were fit to each CpG site separately, and modeled logit-transformed methylation as the response against quartiles of total urinary arsenic (quartile 1 treated as the referent group). Models for U-As were adjusted for maternal age at delivery, infant sex, and urinary creatinine levels. Although our examination was exploratory in nature, *p*-values were adjusted for multiple comparisons by computing the Benjamini–Hochberg adjusted *p*-values (Benjamini and Hochberg 1995). CpG loci that exhibited a linear dose–response relationship were identified by fitting a series of linear regression models, which modeled U-As quartiles as a continuous covariate; U-As quartiles were assigned values of 1–4, for quartiles 1–4, respectively.

All analyses were carried out using the R statistical package, version 2.13 (Vienna, Austria; www.r-project.org/).

## Results

Demographic and clinical information for the 134 mother–infant pairs is provided in Table 1. Home tap-water arsenic concentrations ranged from close to the detection limit (0.03 μg/L) to nearly 100 μg/L, with 22 women (18%) having home drinking water > 10 μg/L, the current U.S. Environmental Protection Agency (EPA) standard (U.S. EPA 2001). Total U-As, calculated by summing the concentrations of inorganic arsenic, MMA^V^, and DMA^V^, ranged from (0.45 μg/L) to nearly 300 μg/L, was highly right-skewed [see Supplemental Material, Figure S3 (http://dx.doi.org/10.1289/ehp.1205925)], and had a median concentration of 4.1 μg/L [interquartile range (IQR), 1.8–6.6], which is similar to the median estimated for the U.S. population as a whole (Caldwell et al. 2009). A single outlying sample with a urinary As value of nearly 300 μg/L was confirmed. Because of the skew of the data and this outlier, as well as the potential nonlinear relationship between arsenic exposure and DNA methylation, we examined total urinary arsenic in all analyses as a categorical variable splitting the data in quartiles.

**Table 1:** Demographic, clinical, and phenotypic information for the study population.

Characteristic	Value
No. of mother–child pairs	134
Maternal age at delivery (years)	31 ± 4.6
Gestational age (weeks)	40 ± 1.3
Birth weight (kg)	3.5 ± 0.46
Infant sex
Male	67 (50)
Female	67 (50)
Delivery type	
Vaginal	92 (69)
Cesarean section	40 (30)
Unknown	2 (1)
Smoking status
Never	97 (72)
Former	11 (8)
Current	4 (3)
Unknown	22 (16)
School level
Less than 11th grade	2 (1)
High school graduate or equivalent	11 (8)
Junior college graduate or some college or technical school	30 (22)
College graduate	48 (36)
Postgraduate schooling	21 (16)
Unknown	22 (16)
Maternal urinary As (μg/L)	4.1 (1.8–6.6)
iAs (μg/L)^*a*^	0.25 (0.13–0.47)
MMA^V^ (μg/L)	0.31 (0.15–0.5)
DMA^V^ (μg/L)	3.4 (1.6–5.7)
Tap-water arsenic (μg/L)	1.2 (0.2–6.2)
Values are presented as mean ± SD, *n* (%), or median (interquartile range). ^******^^***a***^Fifty-six samples had measurements below the limit of detection for iAs.	

Supplemental Material, Table S3 (http://dx.doi.org/10.1289/ehp.1205925) presents coefﬁcient estimates and corresponding *p*-values for quartiles of total U-As and their association with shifts in estimated lymphocyte subsets. Coefficient estimates for each cell type reflect the estimated percent difference in the proportion of that cell type between U-As quartiles 2–4 relative to the lowest quartile (quartile 1). There were no statistically significant overall differences in the proportions of cell types among quartiles of total U-As. Table 2 provides the results of this model fit to continuous values of total urinary inorganic As and metabolites individually where the coefficient estimates represent the estimated change in the proportion of a particular cell type based on a 1-μg/L increase in the levels of these metabolites. For iAs/(iAs + MMA^V^ + DMA^V^), the coefficient estimates represent the expected difference in cell type proportions for a 1-unit increase in the proportion of inorganic As to total U-As. There was a statistically significant positive association between a 1-μg/L increase in inorganic arsenic and the proportion of CD8^+^ T lymphocytes (1.18; 95% CI: 0.12, 2.23) (Table 2). CD8^+^ T lymphocytes also were associated with a 1-unit increase in the ratio of inorganic arsenic to total urinary arsenic (9.11; 95% CI: 0.44, 17.79), but were not significantly associated with other fractions (i.e., MMA^V^ and DMA^V^; Table 2).

**Table 2:** Estimated change in the proportions of leukocyte types (95% CI) by continuous specific urinary arsenic exposure measures.

	Lymphocytes				Myeloid cells	
	CD8^+^ T	CD4^+^ T	NK cells	B cells	Monocytes	Granulocytes
iAs (per μg/L)	1.18 (0.12, 2.23)*	–1.24 (–3.15, 0.68)	–0.11 (–1.83, 1.62)	–0.78 (–1.91, 0.36)	–0.37 (–1.31, 0.56)	1.38 (–2.30, 5.06)
MMA^V^ (per μg/L)	0.93 (–0.30, 2.15)	–0.24 (–2.62, 2.14)	–0.48 (–2.59, 1.62)	–0.68 (–1.88, 0.52)	–0.20 (–1.33, 0.93)	1.15 (0.53, –3.02, 5.32)
DMA^V^ (per μg/L)	0.42 (–0.80, 1.64)	–0.10 (–2.40, 2.20)	–0.37 (–2.14, 1.41)	–0.22 (–1.46, 1.01)	–0.27 (–1.16, 0.62)	0.40 (–3.57, 4.36)
iAs/(iAs + MMA^V^ + DMA^V^)	9.11 (0.44, 17.79)*	–11.82 (–27.66, 4.02)	–2.16 (–14.58, 10.27)	–6.05 (–16.4, 4.27)	–1.81 (–8.20, 4.58)	16.91 (–14.12, 47.92)
All four models were controlled for maternal age at delivery, infant sex, and urinary creatinine. Results are based on fitting the cell mixture deconvolution method separately for each arsenic measure (i.e., iAs, MMA^V^, etc.). Values are coefficient estimates (95% CIs), where the coefficient estimates represent the estimated change in the proportion of a particular cell type based on a 1-μg/L increase in the levels of these metabolites. For iAs/(iAs + MMA^V^ + DMA^V^), the coefficient estimates represent the expected difference in cell type proportions for a 1-unit increase in the proportion of inorganic As to total U-As. *p ≤ 0.05.						

Overall, however, white blood cell distributions explained a relatively small proportion of the variability in patterns of cord blood DNA methylation associated with *in utero* exposure to arsenic (3% for total U-As), so the remaining systematic source of variation was hypothesized to be alterations to the methylome itself in some or all of the cell populations examined. We first used PCA to reduce the dimensionality of the genome-wide DNA methylation data and to investigate the extent to which low-level arsenic exposure *in utero* was associated with genome-wide variability in cord blood DNA methylation in our population. The results of this analysis [see Supplemental Material, Table S4 (http://dx.doi.org/10.1289/ehp.1205925)] revealed no statistically significant association between U-As quartiles and principal components 1–3, which were estimated to account for 20%, 12%, and 9% of the variation in methylation across the array.

**Figure 1:**
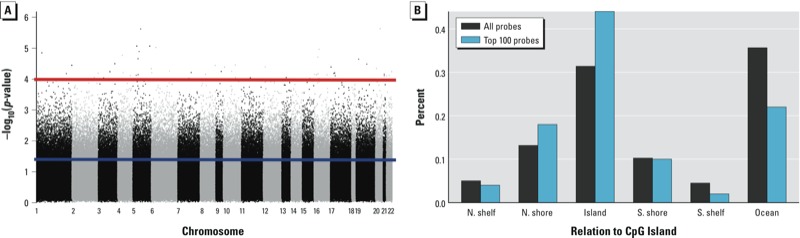
Locus-by-locus examination of differentially methylated CpG sites based on U-As levels. (*A*) Manhattan plot for total U-As, where points represent the -log_10_ (*p*-value) testing the null hypothesis of no difference in methylation across quartiles of arsenic exposure, adjusted for maternal age at delivery, infant sex, and urinary creatinine levels. Red and blue lines indicate –log_10_(1 × 10^–4^) and –log_10_(0.05), respectively. (*B*) Location of the top 100 CpGs associated with U-As on the basis of *p*-value (top 100 probes) compared with all CpGs on the methylation array (all probes). N. shore and N. shelf refer to CpG island shore and shelf regions, respectively, that are upstream of a CpG island region. S. shore and S. shelf refer to CpG island shore and shelf regions, respectively, that are downstream of a CpG island region.

**Figure 2:**
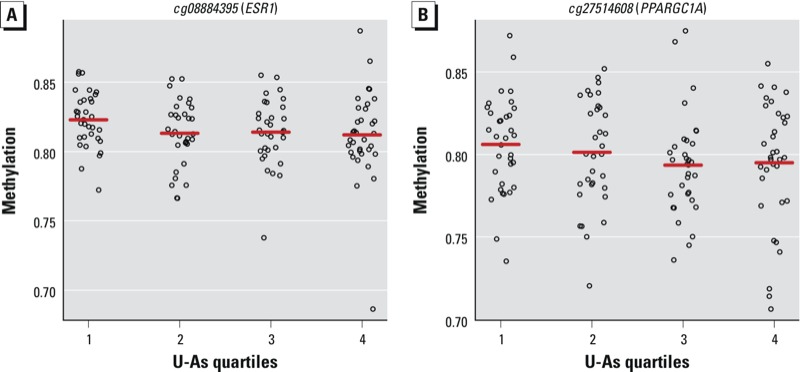
Crude plots of DNA methylation for *cg08884395* (*A*) and *cg27514608* (*B*) by quartiles of total U-As for the 134 study subjects. Red lines denote the within-quartile median methylation status.

To identify individual CpG loci whose methylation status was associated with exposure, we next examined differential locus-specific patterns of DNA methylation based on total U-As levels. As shown in Figure 1A, which depicts –log_10_(*p*-values) (*y*-axis) for the association between U-As quartiles and the methylation of individual CpG loci (the dependent variable), 68,353 of 385,249 CpG loci (18%) were significantly associated with exposure (*p* < 0.05 for a difference over all quartiles of U-As), controlling for maternal age at delivery, infant sex, and urinary creatinine levels. However no association was statistically significant after adjusting for multiple comparisons. Among the 100 CpG loci with the smallest unadjusted *p*-values for the difference in methylation over all U-As quartiles [see Supplemental Material, Table S5 (http://dx.doi.org/10.1289/ehp.1205925)], there was a statistically significantly greater than expected proportion of loci located in CpG Islands (Fisher’s exact *p* = 0.009) (Figure 1B), and of those in CpG Islands (*n* = 44), most (75%) exhibited higher methylation levels in the highest exposed group (quartile 4) compared with the lowest exposed group (quartile 1). Furthermore, a number of differentially methylated CpG loci displayed a linear dose–response relationship across the quartiles of total U-As levels (see Supplemental Material, Table S6). Most notably, our analysis identified *cg08884395* and *cg27514608,* associated with genes *ESR1* (estrogen receptor 1) and *PPARGC1A* (peroxisome proliferator–activated receptor-γ coactivator 1-α), respectively (Figure 2), which both exhibited a negative association between methylation and arsenic exposure (linear trend *p* = 0.0009 for both). Considering the differences in toxicity of inorganic arsenic, MMA and DMA (Le et al. 2000), exposure to different forms of arsenic over the range of U-As concentrations could potentially confound our results. To examine the robustness of our results based on exposure to different forms of arsenic, we refit models to the CpG sites that exhibited a dose–response relationship and individually adjusted for urinary concentrations of inorganic arsenic, MMA, and DMA. These results (see Supplemental Material, Tables S7–S9) indicated that adjustment for urinary arsenic metabolites had very little effect on the association between quartiles of U-As and the DNA methylation of the dose–response CpG sites, with all *p*-values for trend remaining < 0.05.

As validation of the methylation array, two CpG loci (*cg27308738*, *n*=29; and *cg10528424*, *n*=30) were subjected to bisulfite pyrosequencing, because these loci were among the most variable across the study samples in terms of their methylation levels. Our results indicated a very high degree of correlation between the methylation array values and the percent methylation measurements obtained from pyrosequencing (Pearson’s correlation coefficient = 0.98 and 0.93 for *cg27308738* and *cg10528424*, respectively) [see Supplemental Material, Figure S4 (http://dx.doi.org/10.1289/ehp.1205925)].

## Discussion

Patterns of DNA methylation are established during embryogenesis and play an important role in gene transcription, chromosomal stability, X-chromosome inactivation, and tissue differentiation. Alteration of fetal DNA methylation is a potential mechanism linking *in utero* exposures to chronic diseases in adulthood (Kile et al. 2012). The predominantly rural U.S. study population is distinct from prior study populations, which were mainly based outside of the United States and were typically exposed to greater levels of arsenic than those of the study population examined here. However, nearly 20% of this pregnant population was exposed to household drinking water above the U.S. EPA standard. Not surprisingly, most women with household drinking water above the U.S. EPA standard were among those with the highest urinary arsenic levels (data not shown). This is in line with our previous report (Gilbert-Diamond et al. 2011) that arsenic exposure from private well water contributes appreciably to exposure in this population. Our results, which indicated some evidence of differential patterns of DNA methylation across the quartiles of arsenic exposure, support additional investigation into the biologic effects of this level of arsenic exposure and provide further evidence for the need of public health efforts to reduce these exposures.

Animal and *in vitro* studies have demonstrated effects of arsenic at environmentally relevant levels (i.e., 10–100 μg/L) on early immune response (Kozul et al. 2009a, 2009b; Martin-Chouly et al. 2011; Mattingly et al. 2009), providing a potential biological mechanism for health complications experienced by prenatally exposed arsenic individuals later in life. Here we examined whether low-level arsenic exposure at 24–28 weeks gestation, a period during which fetal hematopoiesis is shifting from the liver to the bone marrow (Rodak et al. 2007), was associated with the shifts in immune cells. Our results revealed a statistically significant positive association between the estimated proportion of CD8^+^ T lymphocytes and both inorganic arsenic and the proportion of inorganic arsenic to total urinary arsenic. Although the methylated species of arsenic also exhibited a positive association with CD8^+^ T lymphocytes, unlike inorganic arsenic, the association between MMA and DMA and CD8^+^ T lymphocytes did not meet statistical significance (Table 2). Concentrations of individual metabolites reflect exposure to inorganic arsenic, direct consumption of methylated forms, and methylation efficiency (Kile et al. 2009). Our findings of a stronger association with inorganic arsenic may be attributable partly to the varying toxicity of arsenic’s metabolites or epigenetic activity of arsenic’s metabolites. Thus, our results indicate that increased levels of urinary inorganic arsenic (due to greater *in utero* exposure and/or potentially reduced metabolism of inorganic arsenic to the methylated species) are associated with increased proportions of cord-blood CD8^+^ T lymphocytes. These results are consistent with recent work in which adult mice exposed to 100 μg/L of arsenic over 5 weeks showed increased percentage and total levels of CD8^+^ T lymphocytes (Kozul et al. 2009a). In humans, though, a recent report that evaluated the effects of prenatal arsenic exposure on thymus function at birth in a Bangledeshi cohort of 130 mother–infant pairs raised the possibility that there may be functional deficits in CD8^+^ cells associated with arsenic exposure *in utero* (Ahmed et al. 2012). Although we recognize that our findings are preliminary, we know of no prior studies reporting on relationships between arsenic exposure and cord blood immune cell proportions defined using DNA methylation profiles as a surrogate measure of leukocyte proportion. The potential importance of these findings is underscored by mounting scientific evidence demonstrating that CD8^+^ T lymphocytes contribute to the initiation, progression, and regulation of several pathogenic autoimmune responses (Walter and Santamaria 2005), possibly providing a mechanism by which *in utero* exposure to arsenic during relevant etiologic periods of hematopoiesis results in an increased risk of autoimmune related illness later in life (Tseng 2004). It will be critical to examine prospectively whether the alterations we have observed are linked to immunological consequences later in childhood as well as to validate our findings in additional cohorts.

We further investigated the extent to which low-level arsenic exposure *in utero* was associated with genome-wide DNA methylation variability, assessed using PCA based on DNA methylation in cord blood. This analysis revealed no significant associations between the top three principal components and quartiles of U-As, suggesting that arsenic exposure may not lead to overt, genome-wide perturbations in DNA methylation to the extent that had been suggested by studies of “global methylation” markers. Instead, we found by examining associations between arsenic and each CpG loci individually that 18% of the CpG loci tested were differentially methylated among quartiles of U-As (*p* < 0.05). However none of these associations remained significant after adjustment for multiple comparisons. We observed that the top 100 CpGs associated with total urinary arsenic on the basis of unadjusted *p*-values were disproportionately located in CpG islands, and most of these CpG loci had higher methylation levels in the highest exposed group (quartile 4) compared with the lowest exposed group (quartile 1). Such findings are in line with existing reports, which assert that arsenic exposure is associated with hypermethylation of promoter region CpG islands (Chen et al. 2001) and the well-established role of promoter CpG island methylation in epigenetic gene control and disease states (Ferreira et al. 2012; Lorenzen et al. 2012). Additionally, there were a number of CpG sites that exhibited a linear dose–response relationship with respect to quartiles of U-As [see Supplemental Material, Table S6 (http://dx.doi.org/10.1289/ehp.1205925)], and those relationships remained robust to individual adjustment for the levels of arsenic’s metabolites (see Supplemental Material, Tables S7–S9). Although these results require further validation, they suggest that even low-level *in utero* exposure to arsenic may bring about gene- or CpG-specific epigenetic changes, which themselves may contribute to altered gene expression and downstream cellular function.

In addition to purported effects of arsenic as a carcinogen and immunotoxicant, a growing body of literature is demonstrating endocrine disruption as an additional mode of toxicity (Naujokas et al. 2013). We noted in our analyses that increased levels of total urinary arsenic were associated with decreased methylation of *cg08884395* (linear trend *p* = 0.0009) [Figure 2A; see also Supplemental Table S6 (http://dx.doi.org/10.1289/ehp.1205925)], located in CpG island shore region of *ESR1*, encoding estrogen receptor α. Importantly, methylation of this gene was observed in a CpG island shore region—regions up to 2 kb flanking CpG island—which may be the most enriched with functional CpG sites (Irizarry et al. 2009). In addition, although not statistically significant after adjustment for multiple comparisons, our analysis revealed a decrease in the methylation of *cg27514608*, for increasing quartiles of U-As (linear trend *p* = 0.0009) [Figure 2B; see also Supplemental Table S6 (http://dx.doi.org/10.1289/ehp.1205925)]. This locus is associated with the gene *PPARGC1A*, encoding PGC-1α, a coactivator of several nuclear receptors including peroxisome proliferator-activated receptors α and γ, thyroid hormone receptor, mineral corticoid receptor, and estrogen receptors (Sugawara et al. 2001). The result with *ESR1* is consistent with a report of decreased methylation and increased expression of *ESR1* with *in vitro* arsenic exposure in a breast cancer model (Du et al. 2012), whereas the association with *PPARGC1A* is consistent with a report demonstrating up-regulation of this gene in arsenic-induced Bowen’s disease, a skin carcinoma (Lee et al. 2011). Given the role of hormone receptors and their pathways in fetal development and throughout childhood, and the role that arsenic may be playing in altering these pathways, our data suggest that epigenetic modes of altered regulation of endocrine pathways may also be important for future study.

There are notable limitations to the present study. Our study cohort is largely Caucasian, which could limit generalizability. However, our focus on common exposure levels is unique compared with studies based in arsenic-endemic regions. Although our study did not directly assess concentrations of arsenic on umbilical cord blood as a measure of fetal exposure to arsenic, arsenic is known to readily cross the placenta, leading to fetal serum concentrations similar to maternal levels (Concha et al. 1998; Hall et al. 2007). Also, the external reference set used in our cell mixture analysis, which consisted of DNA methylation signatures from purified white blood cells, was isolated from different, anonymous, adult, nondiseased individuals’ whole blood (Koestler et al. 2012). As differences in DNA methylation have been demonstrated in individuals of varying ages (Winnefeld and Lyko 2012), the white blood cell–specific methylation signatures may not accurately reflect cell-specific patterns of DNA methylation evident in cord blood, which consists primarily of infant blood. Further, our cell mixture analysis used signatures of DNA methylation as a surrogate for cell mixture composition, whereas a complete assessment of the immune proﬁle would require ﬂow cytometric measurements. Although the results from our locus-by-locus analysis showed some evidence of altered methylation with arsenic exposure, these results did not withstand multiple comparison corrections. Thus, these results need to be replicated in additional studies. Along the same lines, we do not have the power to consider differential effects by sex or other potential modifiers, but larger studies should consider such effects.

## Conclusions

Our novel exploratory examination suggests that low-level arsenic exposure *in utero* may influence the infant epigenome. Particularly, our findings highlight the possibility that exposure to arsenic—even at levels common to much of the world’s population—during relevant etiologic periods of fetal development may induce shifts in underlying cell populations, as well as gene-specific alterations in DNA methylation. Long-term follow-up is planned to determine whether the observed changes are associated with short- and long-term health outcomes.

## Supplemental Material

(733 KB) PDF

(98 KB) XLSX

## References

[r1] Ahmed S Ahsan KB Kippler M Mily A Wagatsuma Y Hoque AM 2012 *In utero* arsenic exposure is associated with impaired thymic function in newborns possibly via oxidative stress and apoptosis. Toxicol Sci 129 2 305 314; . 10.1093/toxsci/kfs202 22713597

[r2] Banerjee N, Banerjee S, Sen R, Bandyopadhyay A, Sarma N, Majumder P (2009). Chronic arsenic exposure impairs macrophage functions in the exposed individuals.. J Clin Immunol.

[r3] Barr DB, Wilder LC, Caudill SP, Gonzalez AJ, Needham LL, Pirkle JL (2005). Urinary creatinine concentrations in the U.S. population: implications for urinary biologic monitoring measurements.. Environ Health Perspect.

[r4] Benjamini Y, Hochberg Y (1995). Controlling the false discovery rate–a practical and powerful approach to multiple testing.. J R Stat Soc B Met.

[r5] Caldwell KL, Jones RL, Verdon CP, Jarrett JM, Caudill SP, Osterloh JD (2009). Levels of urinary total and speciated arsenic in the US population: National Health and Nutrition Examination Survey 2003–2004.. J Expo Sci Environ Epidemiol.

[r6] Chanda S, Dasgupta UB, Guhamazumder D, Gupta M, Chaudhuri U, Lahiri S (2006). DNA hypermethylation of promoter of gene p53 and p16 in arsenic-exposed people with and without malignancy.. Toxicol Sci.

[r7] Chen H, Liu J, Merrick BA, Waalkes MP (2001). Genetic events associated with arsenic-induced malignant transformation: applications of cDNA microarray technology.. Mol Carcinog.

[r8] Chen Y Graziano JH Parvez F Liu M Slavkovich V Kalra T 2011 Arsenic exposure from drinking water and mortality from cardiovascular disease in bangladesh: prospective cohort study. BMJ 342 d2431; 10.1136/bmj.d2431 [Online 5 May 2011] 21546419 PMC3088786

[r9] Concha G, Vogler G, Lezcano D, Nermell B, Vahter M (1998). Exposure to inorganic arsenic metabolites during early human development.. Toxicol Sci.

[r10] Davis CD, Uthus EO, Finley JW (2000). Dietary selenium and arsenic affect DNA methylation *in vitro* in Caco-2 cells and *in vivo* in rat liver and colon.. J Nutr.

[r11] Du J Zhou N Liu H Jiang F Wang Y Hu C 2012 Arsenic induces functional re-expression of estrogen receptor alpha by demethylation of DNA in estrogen receptor-negative human breast cancer. PLoS One 7 e35957; 10.1371/journal.pone.0035957 [Online 27 April 2012] 22558281 PMC3338760

[r12] Du P Zhang X Huang CC Jafari N Kibbe WA Hou L 2010 Comparison of beta-value and M-value methods for quantifying methylation levels by microarray analysis. BMC Bioinformatics 11 587; 10.1186/1471-2105-11-587 [Online 30 November 2010] 21118553 PMC3012676

[r13] Ferreira HJ Heyn H Moutinho C Esteller M 2012 CpG island hypermethylation-associated silencing of small nucleolar RNAs in human cancer. RNA Biol 9 881 890; 10.4161/rna.19353 [Online 23 May 2012] 22617881 PMC3495749

[r14] Gamble MV, Liu X (2005). Urinary creatinine and arsenic metabolism. Environ Health Perspect.

[r15] Gilbert-Diamond D, Cottingham KL, Gruber JF, Punshon T, Sayarath V, Gandolfi AJ (2011). Rice consumption contributes to arsenic exposure in US women.. Proc Natl Acad Sci USA.

[r16] Gong G, O’Bryant SE (2012). Low-level arsenic exposure, AS3MT gene polymorphism and cardiovascular diseases in rural texas counties.. Environ Res.

[r17] Hall M, Gamble M, Slavkovich V, Liu X, Levy D, Cheng Z (2007). Determinants of arsenic metabolism: blood arsenic metabolites, plasma folate, cobalamin, and homocysteine concentrations in maternal–newborn pairs.. Environ Health Perspect.

[r18] Harper KN Peters BA Gamble MV 2013 Batch effects and pathway analysis: two potential perils in cancer studies involving DNA methylation array analysis. Cancer Epidemiol Biomarkers Prev 22 6 1052 1060; . 10.1158/1055-9965 23629520 PMC3687782

[r19] Hopenhayn-Rich C, Biggs ML, Smith AH (1998). Lung and kidney cancer mortality associated with arsenic in drinking water in cordoba, argentina.. Int J Epidemiol.

[r20] Houseman EA Accomando WP Koestler DC Christensen BC Marsit CJ Nelson HH 2012 DNA methylation arrays as surrogate measures of cell mixture distribution. BMC Bioinformatics 13 86; 10.1186/1471-2105-13-86 [Online 8 May 2012] 22568884 PMC3532182

[r21] Intarasunanont P Navasumrit P Woraprasit S Chaisatra K Suk WA Mahidol C 2012 Effects of arsenic exposure on DNA methylation in cord blood samples from newborn babies and in a human lymphoblast cell line. Environ Health 11 31; 10.1186/1476-069X-11-31 [Online 2 May 2012] 22551203 PMC3506565

[r22] Irizarry RA, Ladd-Acosta C, Wen B, Wu Z, Montano C, Onyango P (2009). The human colon cancer methylome shows similar hypo- and hypermethylation at conserved tissue-specific CpG island shores.. Nat Genet.

[r23] Johnson WE, Li C, Rabinovic A (2007). Adjusting batch effects in microarray expression data using empirical Bayes methods.. Biostatistics.

[r24] Kile ML, Baccarelli A, Hoffman E, Tarantini L, Quamruzzaman Q, Rahman M (2012). Prenatal arsenic exposure and DNA methylation in maternal and umbilical cord blood leukocytes.. Environ Health Perspect.

[r25] Kile ML, Hoffman E, Hsueh YM, Afroz S, Quamruzzaman Q, Rahman M (2009). Variability in biomarkers of arsenic exposure and metabolism in adults over time.. Environ Health Perspect.

[r26] Koestler DC, Christensen B, Karagas MR, Marsit CJ, Langevin SM, Kelsey KT, et al. (2013). Blood-based profiles of DNA methylation predict the underlying distribution of cell types: a validation analysis. Epigenetics.. http://www.landesbioscience.com/journals/epigenetics/article/25430/.

[r27] Koestler DC Marsit CJ Christensen BC Accomando WP Langevin SM Houseman EA 2012 Peripheral blood immune cell methylation profiles are associated with non-hematopoietic cancers. Cancer Epidemiol Biomarkers Prev 21 8 1293 1302; . 10.1158/1055-9965.EPI-12-0361 22714737 PMC3415587

[r28] Kozul CD, Ely KH, Enelow RI, Hamilton JW (2009a). Low-dose arsenic compromises the immune response to influenza A infection *in vivo*.. Environ Health Perspect.

[r29] Kozul CD, Hampton TH, Davey JC, Gosse JA, Nomikos AP, Eisenhauer PL (2009b). Chronic exposure to arsenic in the drinking water alters the expression of immune response genes in mouse lung.. Environ Health Perspect.

[r30] Kuan PF, Wang S, Zhou X, Chu H (2010). A statistical framework for Illumina DNA methylation arrays.. Bioinformatics.

[r31] Le XC, Ma M, Cullen WR, Aposhian HV, Lu X, Zheng B (2000). Determination of monomethylarsonous acid, a key arsenic methylation intermediate, in human urine.. Environ Health Perspect.

[r32] Lee CH, Wu SB, Hong CH, Liao WT, Wu CY, Chen GS (2011). Aberrant cell proliferation by enhanced mitochondrial biogenesis via mtTFA in arsenical skin cancers.. Am J Pathol.

[r33] Lorenzen JM Martino F Thum T 2012 Epigenetic modifications in cardiovascular disease. Basic Res Cardiol 107 245; . 10.1007/s00395-012-0245-9 22234702 PMC3329881

[r34] Majumdar S, Chanda S, Ganguli B, Mazumder DN, Lahiri S, Dasgupta UB (2010). Arsenic exposure induces genomic hypermethylation.. Environ Toxicol.

[r35] Marchiset-Ferlay N, Savanovitch C, Sauvant-Rochat MP (2012). What is the best biomarker to assess arsenic exposure via drinking water?. Environ Int.

[r36] Marsit CJ, Christensen BC, Houseman EA, Karagas MR, Wrensch MR, Yeh RF (2009). Epigenetic profiling reveals etiologically distinct patterns of DNA methylation in head and neck squamous cell carcinoma.. Carcinogenesis.

[r37] Martin-Chouly C, Morzadec C, Bonvalet M, Galibert MD, Fardel O, Vernhet L (2011). Inorganic arsenic alters expression of immune and stress response genes in activated primary human T lymphocytes.. Mol Immunol.

[r38] Mass MJ, Wang L (1997). Arsenic alters cytosine methylation patterns of the promoter of the tumor suppressor gene *p53* in human lung cells: a model for a mechanism of carcinogenesis.. Mutat Res.

[r39] Mattingly CJ, Hampton TH, Brothers KM, Griffin NE, Planchart A (2009). Perturbation of defense pathways by low-dose arsenic exposure in zebrafish embryos.. Environ Health Perspect.

[r40] Naujokas MF, Anderson B, Ahsan H, Aposhian HV, Graziano JH, Thompson C (2013). The broad scope of health effects from chronic arsenic exposure: update on a worldwide public health problem.. Environ Health Perspect.

[r41] Pilsner JR Hall MN Liu X Ilievski V Slavkovich V Levy D 2012 Influence of prenatal arsenic exposure and newborn sex on global methylation of cord blood DNA. PLoS One 7 e37147; 10.1371/journal.pone.0037147 [Online 25 May 2012] 22662134 PMC3360698

[r42] Pilsner JR, Liu X, Ahsan H, Ilievski V, Slavkovich V, Levy D (2007). Genomic methylation of peripheral blood leukocyte DNA: Influences of arsenic and folate in Bangladeshi adults.. Am J Clin Nutr.

[r43] Rahman A, Persson LA, Nermell B, El Arifeen S, Ekstrom EC, Smith AH (2010). Arsenic exposure and risk of spontaneous abortion, stillbirth, and infant mortality.. Epidemiology.

[r44] Rahman A, Vahter M, Smith AH, Nermell B, Yunus M, El Arifeen S (2009). Arsenic exposure during pregnancy and size at birth: a prospective cohort study in Bangladesh.. Am J Epidemiol.

[r45] Reichard JF, Puga A (2010). Effects of arsenic exposure on DNA methylation and epigenetic gene regulation.. Epigenomics.

[r46] Ren X, McHale CM, Skibola CF, Smith AH, Smith MT, Zhang L (2011). An emerging role for epigenetic dysregulation in arsenic toxicity and carcinogenesis.. Environ Health Perspect.

[r47] Rodak BF, Fritsma GA, Doig K. (2007). Hematology: Clinical Principles and Applications. 3rd ed.

[r48] Selgrade MK (2007). Immunotoxicity: the risk is real.. Toxicol Sci.

[r49] Sugawara A, Takeuchi K, Uruno A, Ikeda Y, Arima S, Kudo M (2001). Transcriptional suppression of type 1 angiotensin II receptor gene expression by peroxisome proliferator-activated receptor-γ in vascular smooth muscle cells.. Endocrinology.

[r50] Tseng CH (2004). The potential biological mechanisms of arsenic-induced diabetes mellitus.. Toxicol Appl Pharmacol.

[r51] U.S. EPA (U.S. Environmental Protection Agency). (2001). National primary drinking water regulations; arsenic and clarifications to compliance and new source contaminants monitoring.. Fed Reg.

[r52] Vahter M (2008). Health effects of early life exposure to arsenic.. Basic Clin Pharmacol Toxicol.

[r53] Walter U, Santamaria P (2005). CD8^+^ T cells in autoimmunity.. Curr Opin Immunol.

[r54] Wilhelm CS, Kelsey KT, Butler R, Plaza S, Gagne L, Zens MS (2010). Implications of LINE 1 methylation for bladder cancer risk in women.. Clin Cancer Res.

[r55] Winnefeld M Lyko F 2012 The aging epigenome: DNA methylation from the cradle to the grave. Genome Biol 13 165; 10.1186/gb4033 22839706 PMC3491376

[r56] Yang HH Hu N Wang C Ding T Dunn BK Goldstein AM 2010 Influence of genetic background and tissue types on global DNA methylation patterns. PLoS One 5 e9355; . 10.1371/journal.pone.0009355 20186319 PMC2826396

[r57] Yunus M, Sohel N, Hore SK, Rahman M (2011). Arsenic exposure and adverse health effects: a review of recent findings from arsenic and health studies in Matlab, Bangladesh.. Kaohsiung J Med Sci.

[r58] Zhao CQ, Young MR, Diwan BA, Coogan TP, Waalkes MP (1997). Association of arsenic-induced malignant transformation with DNA hypomethylation and aberrant gene expression.. Proc Natl Acad Sci USA.

