# Supplementary material for: Differential DNA Methylation in Umbilical Cord Blood of Infants Exposed to Low Levels of Arsenic in Utero
Source: Environ Health Perspect. 2013 Jun 11;121(8):971–7. doi: 10.1289/ehp.1205925 (PMC3733676; doi:10.1289/ehp.1205925)
Supplement: (733 KB) PDF [file ehp.1205925.s001.pdf]

Supplemental Material

Differential DNA Methylation in Umbilical Cord Blood of Infants Exposed to Low Levels of Arsenic *in Utero*

Devin C. Koestler, Michele Avissar-Whiting, E. Andres Houseman, Margaret R. Karagas, and Carmen J. Marsit

Table of Contents:

Methods: Bisulfite Pyrosequencing ……………………………...Page 2

Supplemental Material, Tables S1-S9...…………………..............Page 3 (separate Excel file)

Supplemental Material, Figure S1..……………………………….Page 4

Supplemental Material, Figure S2……………………….............Page 5

Supplemental Material, Figure S3……………………….............Page 6

Supplemental Material, Figure S4……………………….............Page 7

References…………………………..Page 8

Methods: Bisulfite Pyrosequencing

As an orthogonal array validation, two CpG loci (cg27308738, n=29 and cg10528424, n=30) were subjected to bisulfite pyrosequencing, as these loci were among the most variable loci across the study samples. DNA samples isolated using the QIAamp DNA FFPE Tissue Kit (1µg) (Qiagen Inc., Valencia, CA) were bisulfite-modified using the EZ DNA Methylation Kit (Zymo Research, CA, USA). Bisulfite pyrosequencing was performed as previously described (Wilhelm-Benartzi et al. 2012) on the Pyromark MD Pyrosequencer (Joubert et al. 2012). Results were analyzed using the PyroMarkCpG software version 1.0.11 (Qiagen) to obtain the methylation percent at target CpG loci. Amplification of bisulfite converted DNA was accomplished using the PyroMark PCR kit (Qiagen) and forward and reverse biotinylated primers (IDT Inc., Coralville, IA) for each target loci. Cycling conditions were 95 °C for 15 min. followed by 45 cycles of 94°C for 30 sec., 60°C for one min. and 72°C for one min. with a final extension of 10 min. at 72°. Pyrosequencing reactions were performed in triplicate for each sample and the mean of the triplicates used in subsequent analyses. All pyrosequencing reactions included bisulfite modification controls and >93% modification efficiency was accomplished for all samples included in the analysis.

__Supplemental Material, Tables S1-S9:__
Supplemental Material, Tables S1-S9 are contained in a separate Supplemental Material file as separate Excel worksheets. Legends for Tables S1-S9 are provided below. Footnotes are provided on the individual table worksheets.
NOTE: All *EHP* content is accessible to individuals with disabilities. To request fully accessible (Section 508–compliant) versions of the supplemental tables, please go to http://ehp.niehs.nih.gov/accessibility/.

Supplemental Material, Table S1: Results examining the association between the top three principal components (PCs), derived from the raw/unadjusted cord-blood DNA methylation data, and various technical, clinical and patient level covariates. PC_p represents the pth principal component with (%) representing the percent variability in genome-wide methylation explained by that PC.

Supplemental Material, Table S2: Results examining the association between the top three principal components (PCs), derived from the plate-adjusted cord-blood DNA methylation data, and various technical, clinical and patient level covariates. PC_p represents the pth principal component with (%) representing the percent variability in genome-wide methylation explained by that PC.

Supplemental Material, Table S3: **Results from the cell mixture analysis applied to total urinary arsenic levels.** Values are expressed as, coefficient estimate (CI). Coefficient estimates in Table S3 reflect the expected difference in cell type proportions between quartiles 2, 3, and 4 and the referent group (quartile 1).

Supplemental Material, Table S4: **P-values obtained from examining the association between quartiles of U-As and the first 3 principal components (PCs).** PC_p represents the pth principal component with (%) representing the percent variability in genome-wide methylation explained by that PC.

Supplemental Material, Table S5: Top 100 CpG loci associated with quartiles of U-As on the basis of overall P-value. Overall P-value refers to the p-value obtained from a hypothesis test of no difference in methylation across quartiles of U-As.

Supplemental Material, Table S6: CpGs that exhibited a dose-response relationship with respect to quartiles of U-As.

Supplementary Material, Table S7: CpGs that exhibited a dose-response relationship with respect to quartiles of U-As, upon adjustment for levels of inorganic arsenic.

Supplementary Material, Table S8: CpGs that exhibited a dose-response relationship with respect to quartiles of U-As, upon adjustment for levels of MMA.

Supplementary Material, Table S9: CpGs that exhibited a dose-response relationship with respect to quartiles of U-As, upon adjustment for levels of DMA.

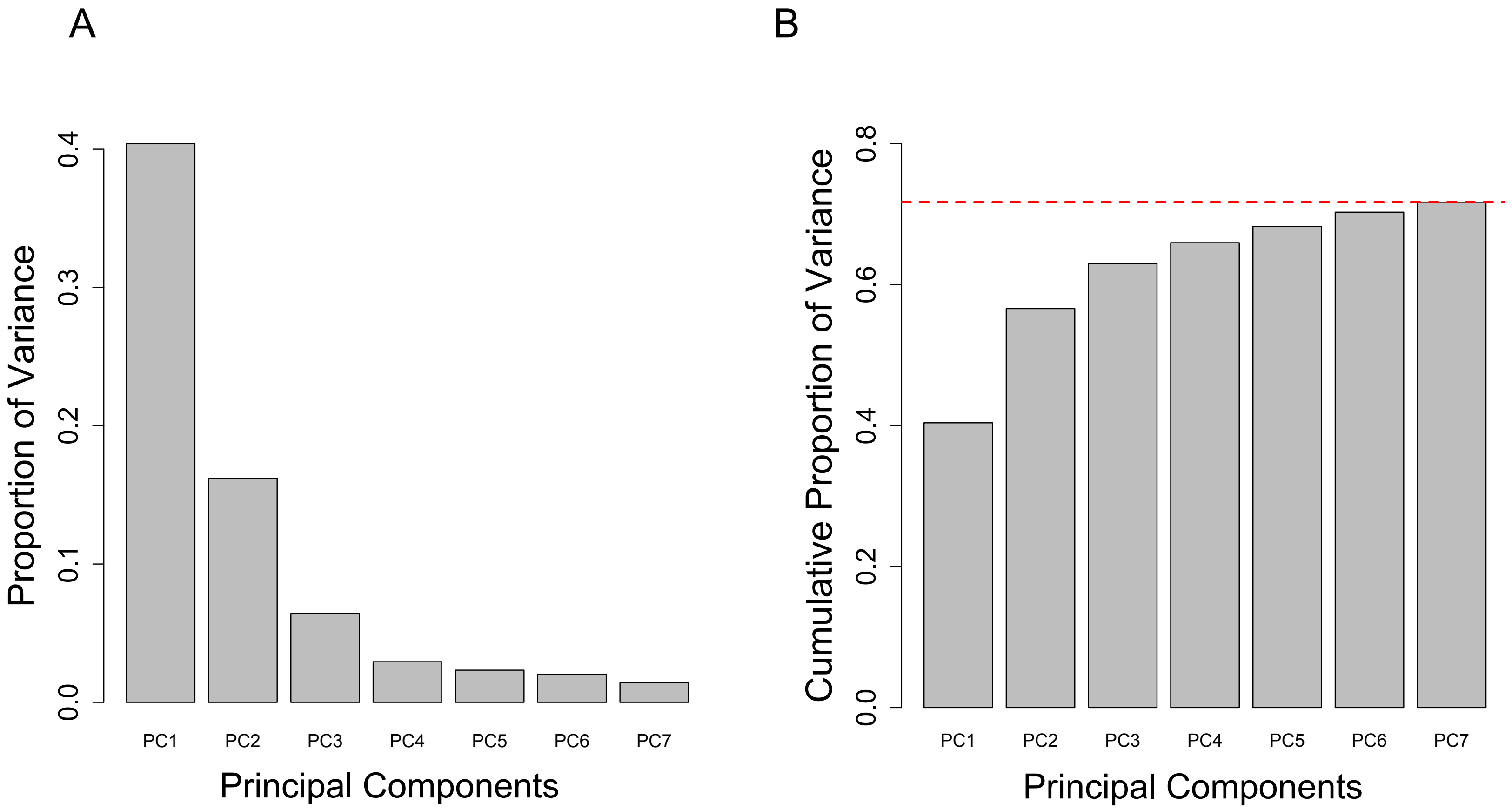

Supplemental Material, Figure S1: Plots of the proportion (A) and cumulative proportion (B) of variance explained by the first seven principal components, derived from the raw/unadjusted cord-blood DNA methylation data. Raw/unadjusted refers DNA methylation data refers to data that is unadjusted for plate-effects. Figure S1 shows that the top 3 principal components accounted for approximately 65% (panel B) of the variability in methylation for the raw, non-plate adjusted methylation data.

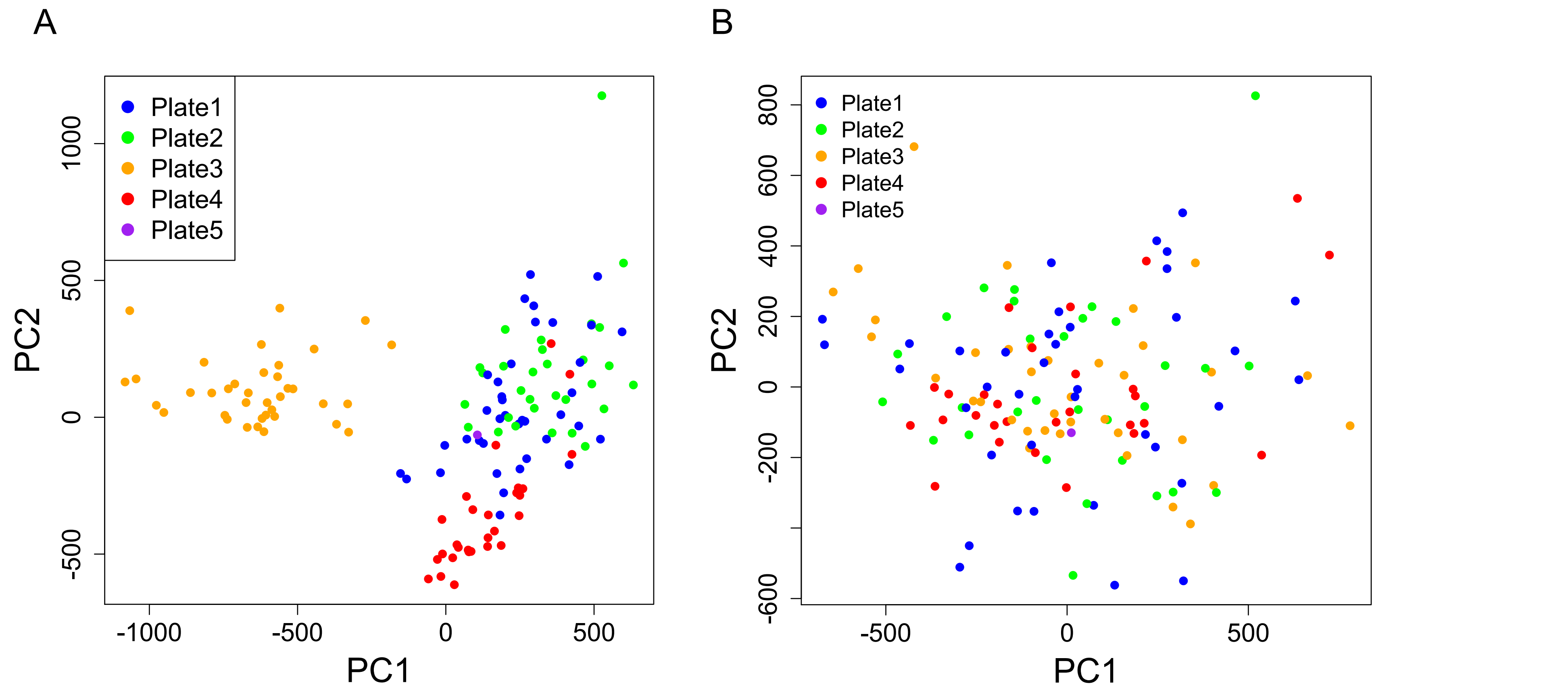

Supplemental Material, Figure S2: Plots of the first two principal components extracted from (A) raw/unadjusted DNA methylation data (B) plate-adjusted DNA methylation data. Colored dots indicate the 5 different plates from which the DNA methylation data was obtained. Figure S2 (A) shows a strong clustering pattern of plate based on the first two principal components derived from the raw, non-plate adjusted DNA methylation data, suggesting that plate-effects account for a substantial fraction of the variability in DNA methylation. Figure S2 (B) indicates that ComBat procedure was successful in attenuating the effects of plate on variability in DNA methylation.

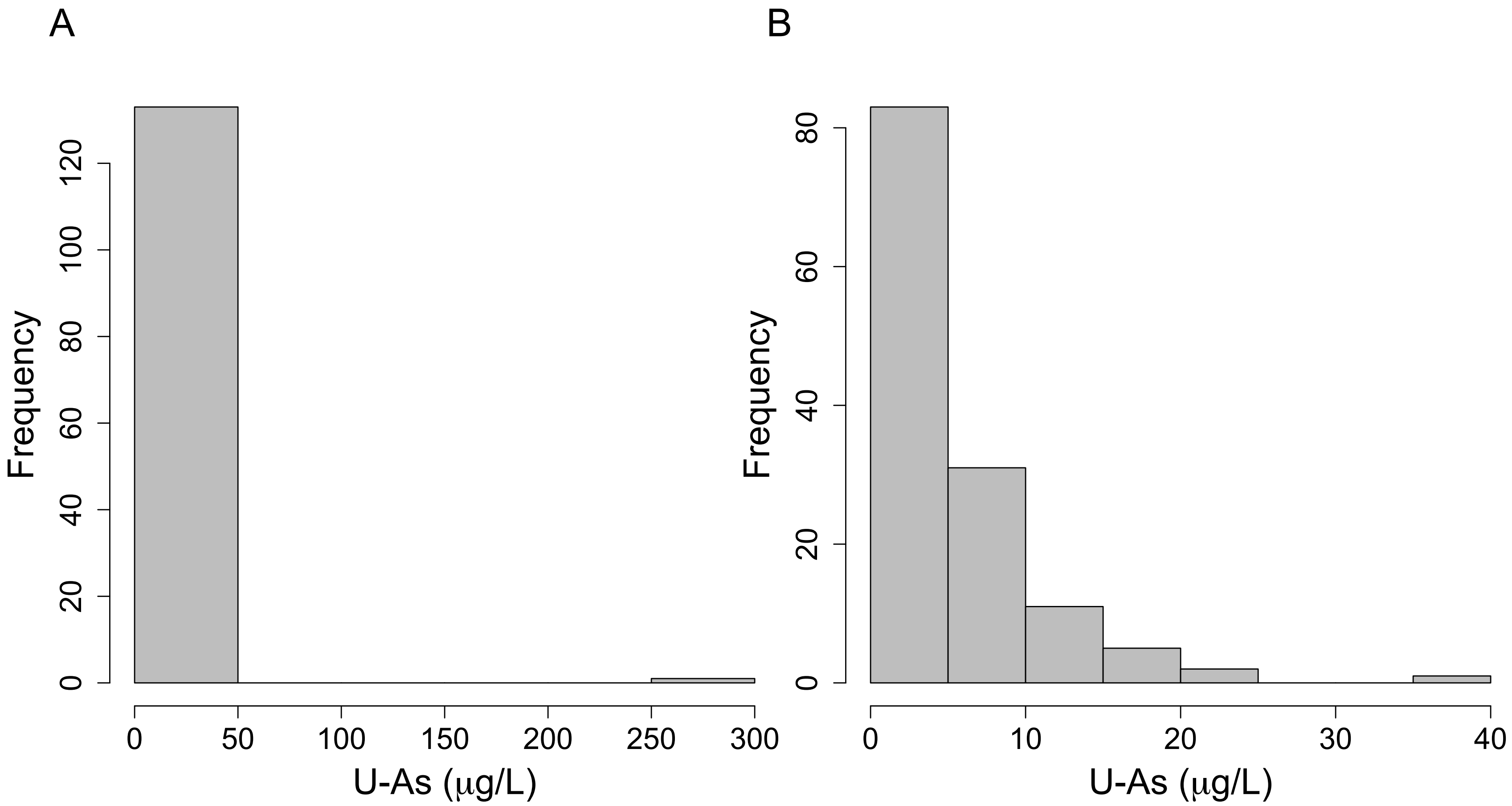

Supplemental Material, Figure S3: Distributions of total urinary arsenic (U-As) expressed as μg/L, with (A) and without (B) an outlying sample.

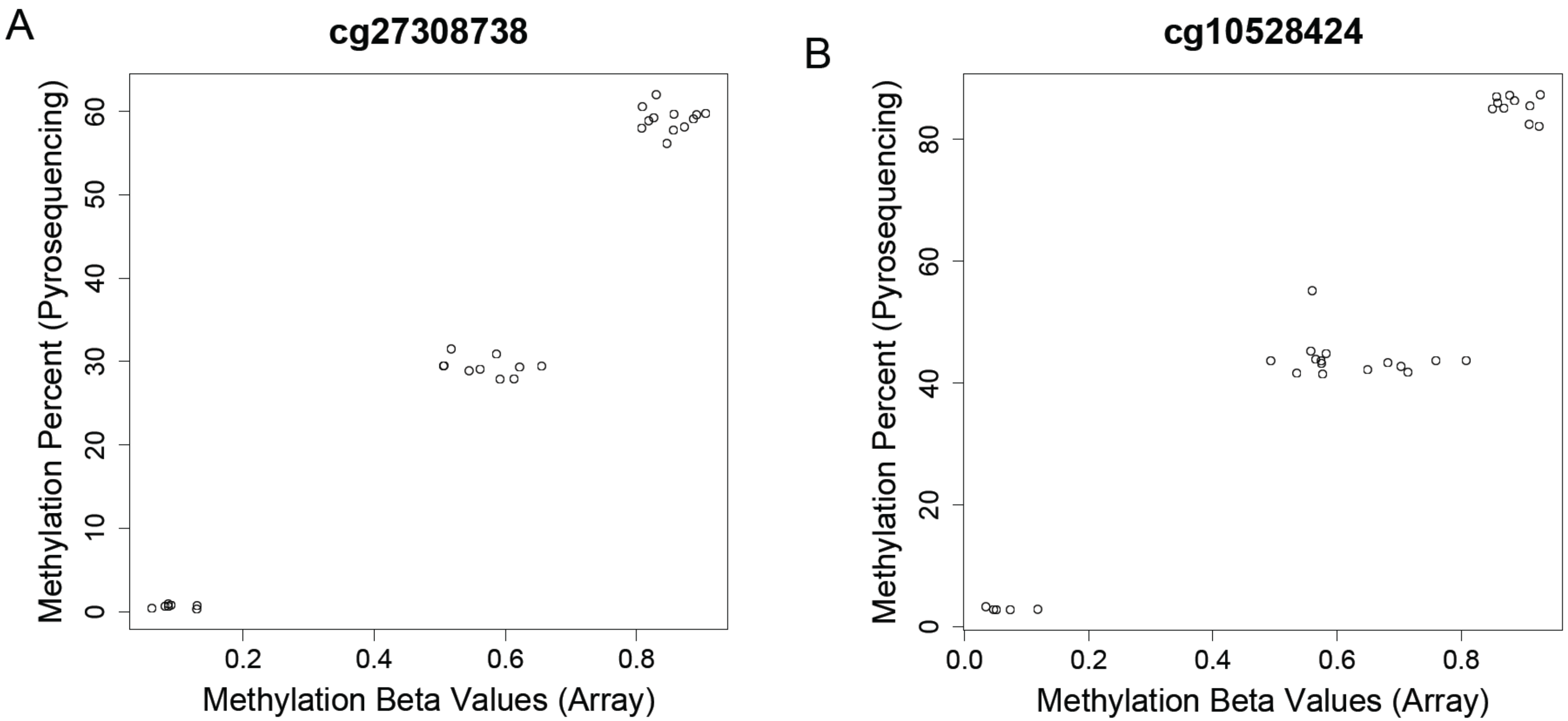

Supplemental Material, Figure S4: Scatterplots of the methylation percent via bisulfite pyrosequencing and array methylation beta values for (A) cg27308738 and (B) cg10528424

References

Joubert BR, Haberg SE, Nilsen RM, Wang X, Vollset SE, Murphy SK, et al. 2012. 450K Epigenome-Wide Scan Identifies Differential DNA Methylation in Newborns Related to Maternal Smoking during Pregnancy. Environmental health perspectives 120(10): 1425-1431.

Wilhelm-Benartzi CS, Houseman EA, Maccani MA, Poage GM, Koestler DC, Langevin SM, et al. 2012. In utero exposures, infant growth, and DNA methylation of repetitive elements and developmentally related genes in human placenta. Environ Health Perspect 120(2): 296-302.
